# Collectively learning to talk about personal concerns in a peer‐led youth program: A field study of a community of practice

**DOI:** 10.1111/hsc.13844

**Published:** 2022-05-24

**Authors:** Niels Buus, Maja Moensted

**Affiliations:** ^1^ Faculty of Medicine, Nursing and Health Sciences Monash University Melbourne Australia; ^2^ Department of Regional Health Research University of Southern Denmark Odense Denmark; ^3^ Sydney School of Health Sciences, Faculty of Medicine and Health University of Sydney Australia; ^4^ Central Clinical School, Discipline of Addiction Medicine, Faculty of Medicine and Health University of Sydney Australia

**Keywords:** communities of practice, community intervention, high‐risk populations, peer‐learning, young adulthood

## Abstract

Youth peer‐support is an increasingly established approach to youth work. Drawing on Wenger's theory of a ‘community of practice’, this study explored social learning processes in an Australian community‐based and peer‐led youth program for young people from disadvantaged backgrounds. Social learning was conceptualised as arising from processes of ‘negotiated meaning’, which Wenger described as a duality of ‘participation’ and ‘reification’. The study was designed as a qualitative field study drawing on participant observation data from weekend workshops and 16 semi‐structured interviews with young program participants. Results indicate that the program was highly conventionalised with repeated practices reifying the meaning negotiated by the participants. However, it was also open for participatory negotiation of meaning through which participants learned to recognise and share their own and others' vulnerabilities, which created a strong sense of belonging to a community of equals. The study's conception of social learning offers a novel explanation of how the youth program created and sustained alternative transformative spaces. This was done through simple, repetitive and highly conventionalised practices, brokered by peers, that allowed participants to recognise and address their own and others' vulnerabilities in new ways.

**What is known about this topic?**

- Youth services are increasingly criticised for being sites of disempowerment of young people.
- Peer support can be a crucial resource for disadvantaged young people.

**What this paper adds?**

- Highly contextualised insight into the social learning processes generating social support for—and between—young people with disadvantage.
- A demonstration of the fruitfulness of Wenger's conception of ‘negotiated meaning’, most notably ‘participation’ and ‘reification’.

## 1 INTRODUCTION

A central aim of youth work is to facilitate alternative psychosocial support contexts for young people from disadvantaged backgrounds to ultimately reshape their perceptions of possibilities (Moensted, 2018) and to assist them altering their own trajectories through life and in becoming more active participants of society (Bessant, 2019). However, this is often impeded by structural inequities and limited psychosocial and material resources to engage with conventional helping systems. Moreover, young people from disadvantaged backgrounds frequently have experiences of exclusion and low achievement in mainstream educational settings (Moensted, 2022), in which learning and participation often are based on middle‐class values (Miles, 2007). A key challenge for many youth programs is therefore to offer acceptable alternatives to conventional school‐based settings that can create different learning experiences for young people (McGregor, 2017).

Some educational youth programs have specialised in developing learning environments with relatively conventional curricula and targets for the development of competencies, such as after‐school homework assistance programs. Other programs have specialised in developing learning experiences while engaging with non‐conventional curricula, for example through the use of arts and drama, see for instance (Cahill, 2013; Wright et al., 2016). The aim of the latter programs includes the development of different learning experiences through the recognition of capabilities and the fostering of trust and self‐worth, which may be used by the young person in other life/learning situations.

Over the past decade, there has been greater attention placed on the benefits of peer support models within youth work and a robust theoretical basis for the value of peer support programs has been established (Bassuk et al., 2016; Cohen & Wills, 1985; Dolan & Brady, 2012; Paquette et al., 2019). Peer support has been identified as a crucial resource for disadvantaged young people (DuBois et al., 2002), as such support models positively affect young people's self‐esteem, self‐efficacy and perceived locus of control (Turner, 1999). Additionally, peer support has been found to be empowering and beneficial for both users and those involved in providing such support (Turner & Shepherd, 1999). The positive role modelling and sharing of information and guidance by peers are often more successful than support offered by professionals, as young people identify with their peers (Buehlmann, 2010). The support of peers has been found to assist vulnerable and disadvantaged young people by reinforcing positive, and reducing risky, behaviours and cultivating new alternative behaviours (Buehlmann, 2010; Moensted & Buus, 2022).

Despite the well‐establish knowledge about the benefits of peer support for young people experiencing personal challenges, more knowledge is needed on how such support models can be strengthened and tailored to suit the needs of disadvantaged young people. One way of developing such knowledge is through detailed explorations of young people's learning processes as they participate in peer‐support, and in this paper, we examine a peer‐led youth program in Australia, which did not follow a conventional curriculum.

### 1.1 Theoretical perspective: Social learning in communities of practice

We explored the collective learning processes in a youth program from a social learning perspective (Lave & Wenger, 1991), which views learning as inseparable from social practices. More specifically, we conceptualised young people's participation in shared practices at a youth program as forming a ‘community of practice’ (Wenger, 1998), where young people learn together and become legitimate and gradually more central community members (Lave & Wenger, 1991).

‘Learning in practice’ is therefore concerned with people's ability to engage in shared practices, their shared development of understanding of why they engage in it, and the shared resources they have at their disposal to negotiate them (Wenger, 1998). According to Wenger (1998), active participants in shared practices can generate collective learning, which can form the particular social configurations named ‘communities of practice’ and inform participants' identities. Learning arises from processes of ‘negotiated meaning’, which is described as a duality of ‘participation’ and ‘reification’ (Wenger, 1998). ‘Participation’ is meaning‐making social action that shapes people's experiences and form communities and community membership. However, as meaning‐making will always to some extent be constrained by its historical and social situatedness, ‘reification’ refers to processes and outcomes that inform and shape experienced meaning.

While Wenger's theory on learning through participation in communities of practice has had a significant influence on the fields of sociology and education, it has been taken up to a lesser degree within youth studies. Buccieri (2010) described a community of practice of marginalised youth and service providers practicing harm‐minimisation. Goodnough (2014) explored a youth‐led community of practice coalescing around an action research project. Both Buccieri (2010) and Goodnough (2014) utilised Wenger's (1998) theory of communities of practices to identify and describe the formation and character of partnerships. Others have instead focused on identifying the formation of a community of practice in actual moment‐to‐moment language interactions.

Miles (2007) described a performance‐based learning project for excluded youth as the formation of a community of practice. Bonnette and Crowley (2020) described how three ‘emancipated emerging adults’ engaged with a ‘Makerspace’ community of practice through legitimate peripheral learning. The studies by Miles (2007) and Bonnette and Crowley (2020) argue that the shared creative practices of the observed communities of practice had the potential to assist young people in taking personal ownership of their learning experience and learning to express themselves through creative achievement, which was not possible in mainstream education drawing on conventional curricula.

The previous studies from youth studies often share a tendency to identify and verify the key elements of Wenger's theory, such as legitimate peripheral participation, joint enterprise, mutual engagement and shared repertoire. This relatively limited use of the theory is not an uncommon observation (Smith et al., 2017). In the current paper, we will emphasise one of Wenger's (1998) less utilised ideas concerning the relationship between ‘participation’ and ‘reification’, which can open up original and detailed analyses of collective learning processes in a community of practice.

The aim of this study was to explore ‘participation’ and ‘reification’ in collective learning processes in a peer‐led youth program for disadvantaged young people in Australia.

## 2 MATERIALS AND METHODS

This study was designed as a qualitative field study (Hammersley & Atkinson, 2019) drawing primarily on participant observation data from weekend workshops and data from semi‐structured interviews with young program participants, and secondarily on contextual data collected as part of a larger evaluation of the program (Moensted & Buus, 2022; Moensted et al., 2020). We chose the study design, because it could yield nuanced and contextualised insight into the key characteristics of the youth program's social practices, including relationships between participation and reification.

### 2.1 Study context

The study context was a peer‐led youth program targeted socio‐economically disadvantaged young people aged 14 to 20. The program is run by a not‐for‐profit organisation, Youth Insearch, which operates across three Australian states, New South Wales, Queensland and Victoria. Youth Insearch is a relatively inclusive program that does not specify the type or level of ‘disadvantage’, but seeks to relieve young people's experiences of suffering and helplessness. The program was established in 1985 following a co‐design process between a social worker and his young clients. At the core of the program are community support, including weekly support groups, peer‐to‐peer support, mentoring and weekend workshops. Weekend workshops were located away from the young people's usual settings with the aim of ‘short circuiting’ their problem definitions and as well as their sense of possible solutions.

The program involves young people and adults in both waged and volunteer roles. Through internal leaders training and mentoring (Douglas et al., 2019), Youth Insearch invites selected and engaged young people and adults to take up leadership roles in the program and become ‘young leader’ or ‘adult leader’. The peer‐focus emphasises the role young people play in determining, creating and applying change processes for their peers, and young leaders are positioned to share guidance and offer advice to program participants, which adds to a shared sense of new possibilities and hope (Moensted & Buus, 2022). In an earlier evaluation, the program was interpreted as parallel to a therapeutic community (Ghayour‐Minaie & Toumbourou, 2016).

### 2.2 Data collection

Two ethnographic field workers generated data between December 2019 and March 2020 when they were participant observers at a variety of program practices. Both researchers were middle‐aged, one identifying as male and the other as female, and had extensive research experiences in the sociology of health and illness and the sociology of youth. Data were generated on the basis of field notes (Emerson et al., 2011), which included writing scrap notes at the program as well as extensive descriptions and theoretical/personal reflections developed post hoc. Participant observation also included informal interviews with program participants and young and adult volunteers, and staff during weekend workshops. Data were also generated on the basis of formal interviewing of 16 young program participants (Holstein & Gubrium, 1995) that lasted between 45 and 120 min with an average length of 60 min. These were audio‐recorded and transcribed verbatim by a professional typist. These interviewees were 16.25 years old on average; 50% identified as Australian and 50% as aboriginal; and eight identified as female, seven as male, and one as non‐binary. Finally, as part of the wider evaluation, we generated contextual data by formally interviewing young and adult volunteers and staff (Moensted & Buus, 2022; Moensted et al., 2020).

### 2.3 Data analysis

The analysis was a process of open coding of the data set and extensive memo‐writing (Emerson et al., 2011). The participant observation was both collaborative and independent, which allowed us to discuss both shared and individual field experiences. We both coded the data independently before discussing our emerging interpretations. This led to the identification and description of a range of program happenings and events as well as patterns in the participants' perspectives. After a period of extensive discussions where we considered different theoretical perspectives on the data set and the preliminary analysis, we decided to use Wenger's (1998) social learning perspective on community and identity formation. We anticipated that an analysis from this perspective could emphasise learning in the program and of youth work more generally that have hitherto been under‐reported. Wenger's concepts were used to focus the analysis and the findings reported here. The final part of the analysis consisted of a systematic judgement of ‘trustworthiness’ with a particular emphasis on ‘credibility’ (truth value) and ‘dependability’ (consistency) of the findings (Guba, 1981).

### 2.4 Ethics

The University of Sydney Human Research Ethics Committee approved this study. All weekend workshop participants were informed about the research and all interview respondents gave written consent to participate in the study. To protect the identities of the participants, all names in the data presentation were replaced with pseudonyms and potentially identifying details from the participants' narratives have been altered or omitted.

## 3 RESULTS

The findings are thematically organised under four headings emphasising and exploring processes of participation and reification in workshops practices. (1) Reification in the negotiation of meaning in group practices, (2) Participation in the negotiation of collaborative physical practices, (3) Reification in shared storytelling and (4) Participation in the negotiation of the meaning of sharing personal experiences.

The weekend workshops were organised around a number of shared practices that were informally described as young people ‘coming together to talk about their issues’. Their participation formed a community of practice of young people concerned with very heterogeneous issues, that is to say, the young people were experiencing a mix of contextual and individual challenges, such as residing within families or foster homes marked by multiple social problems (such as alcohol and drug use, poverty, violence, psychiatric problems and unemployment) and few social and material resources. Most respondents described their experiences from their first workshops in terms of significant personal change, which for many was reported as a life‐altering conversion experience.

### 3.1 Reification in the negotiation of meaning in group practices

A significant part of the practice of ‘coming together to talk’ was concerned with identifying oneself in the big group, through which highly structured and conventionalised practices reified collective learning processes. The participants would come together as a big group in a large space with a chair for each of the approximately 80 participants, out of which half were young participants, and the other half young leaders, adult leaders, volunteer support adults and waged staff. The chairs were placed close to each other in a big circle, and as all talk took place through a microphone and loudspeakers, all of the seated participants had a good opportunity to see and hear everybody else.

Most study respondents spontaneously reflected on the first time they shared in the big group. Early in a weekend workshop, participants were asked to introduce themselves to the group by stating their name, where they were from and what their favourite film or TV‐show was. This meant that a moderator passed around the microphone in the circle, and as it took place in order, most participants had opportunity to prepare their response. This practice may on the surface seem basic, but was in effect a highly structured exercise in the group's shared practices that hinted at the participant's personal identity to the group. Occasionally, a participant would choose not to respond, and the moderator would encourage a response without insisting if it appeared too challenging for the person. The use of the microphone supported the practice of only one person speaking at a time and kept the pace of talk slow as speakers had to wait for the microphone before talking.

High levels of reification were also conspicuous in the repeated structure of some of the big group sessions, where one or two young leaders moderated the session based on having memorised the presentation. At each session, largely the same questions would be posed, for instance, in the communication session the moderator would ask, ‘Can anyone give an example of communication?’ While the repeats of these sessions frustrated some participants, others acknowledged that while the sessions could feel very repetitive, they were always slightly different because they included different people giving different answers. As observers of the repeated practices, we felt that the structured and repeated sessions created a sense of competence, confidence and safety for recurring participants who would not usually engage in such group practices. Stating your name or remembering an earlier session and responding, for example to the question above about communication, ‘A text message’ can be regarded as a first but significant step towards fuller engagement in the community practices and the formation of ‘legitimate, peripheral participation’ (Lave & Wenger, 1991) and community membership.

For most participants, the reifying big group practices meant that they were very quickly able to safely participate in talking, which for many constituted an interrupted space compared to what they had experienced in other contexts of their lives. These conventionalised practices meant that they experienced that everybody was allowed to speak, and when they spoke, people, both in and out of the big group setting, accepted and recognised speakers for what they shared.

### 3.2 Participation in the negotiation of collaborative physical practices

The practices of ‘coming together to talk’ extended beyond talking in the big group. Music was used to signal the start of a session, but also as a way to thematise and emotionally charge the topics spoken about, for instance, playing Lady Gaga's ‘Till it happens to you’ in the session about sexual abuse. Practices also involved physical touch with the other participants, and the music was often combined with the big group holding hands. Hand holding prevented participants from leaning forward or curling up on the chair, and instead encouraged participants to reach out left and right, open up their chest and connect with the other group members' faces, gazes and bodies in the circle. This further served to create a sense of going through experiences together, interrupting the sense of isolation that some might experience. Some chose to keep the other at an arm's length by stretching their arms out, although most held hands in more relaxed postures.

Another practice was hugging. On the first evening of the weekend workshop—after an introduction to ‘safe’, non‐intrusive and non‐sexual hugging—the participants were placed in a circle of pairs facing each other, and then encouraged to hug each other. After the hug, the inner circle of people would step sideways, and everybody would get a new hugging partner. Each person would hug up to 20 different people in a row. Hugging felt very intense because it took place in silence and participants would look each other in the eye before stepping forward and leaning into the other person's chest and shoulders, body heat and odours. People on the outer circle could see all the other participants hugging. For instance, Ash (participant #2, 16 years old, male, aboriginal, >10 weekend workshops), described hugs as ‘the best experience about the whole camp’ because of a feeling of love and support from the whole room.

While the majority of participants ‘loved’ the hugs, many newcomers found it transgressive at first. An interview participant, Maria, who had attended workshops over a 2‐year period reflected on the first time she was in a hugging session:

‘The first time that we did the hugs, I did not want to participate. But once they reassured me that it would be safe, hugging, it kind of gave me that sense of reassurance that I needed. And it feels really good. (Maria, participant #1, 18 years old, female, Australian, >15 weekend workshops).

The quote highlighted the collaborative practice. Maria was refusing to participate, but she received a ‘brokering’ (Wenger, 1998) reassurance from others to learn safe hugging. On the surface, learning to hold hands and hug someone could be regarded as simple, everyday life activities, but on‐the‐spot negotiating of shared boundaries, tolerating the physical proximity of other people, expressing care and affection and relying and trusting other people in the group were very challenging activities for many of the participants to whom these practices were largely foreign.

### 3.3 Reification in shared storytelling

Telling and listening to stories was a central part of the community's shared practices. A repeated story was the story of the program, that a disgruntled social worker decided to involve a group of young people in co‐designing their own group‐based program, and that the current program was what they produced decades ago. The story highlighted the ingenuity of the social worker and introduced a group of anonymous young people, which the current participants were invited to join and align with through their engagement.

Another repeated story, in support groups and on weekend workshops, was the interactive ‘heart on the wall’, which started out with the storyteller drawing a big heart on a whiteboard and inviting the group to name a newborn's qualities, such as ‘innocence’, ‘ambition’ ‘curiosity’ and more. The group's responses were written into the heart and the storyteller told that as the child grows, situations and people influence the child and close off the child's innate capabilities. Bad influences were listed and represented on the whiteboard as a wall of bricks around the heart. The bricks around the heart were labelled: ‘crime’, ‘drugs’, ‘eating disorders’ and more.

‘The experienced storyteller asked the group if someone feels ‘hrr’ [growled to signify inner pain] and rubbed her fist on her chest. Many of the participants sat with frozen facial expressions as she continued by asking, ‘Do you recognise it? The idea is to tear down the wall this weekend’. The ambition is to re‐discover the innocent little heart. ‘Which brick are you going to work with this weekend?’ and the participants gave suggestions in the big group, their personal goals and thoughts’. (Fieldnote).

The narrative was designed to address the heterogeneity of psychosocial disadvantage and was co‐told by the participants, while encouraging the participants to work on improving their own situation. Represented by the heart, the young people were interpellated to position themselves as innocent victims of events, represented by the bricks in the wall, outside their control. While the plot reified the negotiated meaning, the practice of co‐telling the story allowed the participants to imagine their personal story as part of the bigger, empowering community story.

On the evening of the second workshop day, the big group convened in the group room with the main lights switched off. An initiation ritual followed, where each of the weekend workshop first‐timers were wrapped in a donated knitted blanket, and an adult staff member told a story about refugee families in hiding who would talk as they passed round a candle in the dark. A candle (and a microphone) was passed around in the circle and the vast majority of participants relayed how they felt in that exact moment or shared gratitude and appreciation. Several young people cried quietly during this intimate and caring activity. While the plots in the shared narratives reified meaning, the meaning of stories was open for social interpretation through participation and could form participant identities (Wenger, 1998).

### 3.4 Participation in the negotiation of the meaning of sharing personal experiences

The practice of ‘giving advice’ was frequently discussed on weekend workshops and during interviews. It subsumed a number of ways of responding to someone's story, for instance, acknowledging an experience as shared, sharing what had worked for someone having had similar experiences, and suggesting possible ways forward for someone else.

On the second and third day of a weekend workshop, the sessions would include ‘hassle’ sessions that focused on more challenging topics, such as ‘parent‐adolescent’, ‘grief’ and more. These sessions often focused on a smaller number of participants who would share about their problems in the big group context. These sessions could be very emotionally charged with the person at the center and the witnessing participants in the big circle in tears. Witnessing sometimes included participants in the outer circle sharing similar stories, giving advice and offering support to the person at the center. This practice of talking and sharing served to blur the lines between being in need of support and providing support (Moensted & Buus, 2022). The program included events with both social and personal significance, these events included the first time a young person ‘shared’ (in particular in the big group), and the first time a young person got overwhelmed and cried on weekend workshop. While interview respondents spoke about these big group events, the sharing continually took place in the informal interactions outside structured sessions. A respondent, Ian, was interviewed on the third day of his first‐weekend workshop. He had previously refused to participate in the program practices that involved physical touch. He reflected on how it felt to ‘open up’ and tell his story to some of the other participants after hearing another participant sharing that her mum had cancer:

‘Ian: I only opened up today about my mum having lung cancer, and that was pretty hard, to actually say it. But like, today actually hasn't been that bad, but the other days were really dog shit, like I did not like it at all. I thought everyone was weird. I didn't understand it and it made no sense to me on why everyone would be so open about their personal life. I haven't told anyone about anything that's happened to me in like, six years. I haven't cried in like, five years. So yeah.

Interviewer: So, what happened to you when you spoke about it this morning?

Ian: [laughs] I cried.

Interviewer: You cried?

Ian: Yeah, and that's the first time I've cried in like, six years. It felt shitty actually. It did not make me feel better, it made me feel worse’. (Ian, participant #16, 18 years old, male, Australian, 1stweekend workshop).

Ian had struggled making sense of the program practices of talking openly about personal issues, but he had eventually engaged in this practice and had shared and cried about his mother having a life‐threatening disease. It had made him feel worse at the time, but later in the interview, he described it as a relieving experience.

The structured weekend workshop program, with its repeated practices of teaching/awareness raising and its safe sharing of personal issues amongst peers, reified some of the potential meaning of the practices and created opportunities for the young participants to negotiate meaning and learn together though their participation in ‘coming together to talk’. We suggest that opportunities for learning were augmented by the participants practicing ‘talking together about personal issues’ and, at the same time, rehearsing different modalities of ‘talking together’, including sharing, giving advice and touching. This could be a massively overwhelming experiences for some participants, but for many participants this added to a strong sense of ‘belonging’ (Wenger, 1998) to the ‘Youth Insearch family’ that many participants referred to and which allowed them to create new images of themselves and the world (Moensted & Buus, 2022).

## 4 DISCUSSION

Based on Wenger's (1998) social learning theory, the analysis adds to the growing literature on peer‐support, as it offers an explanation of how psychosocial support is generated on the basis of observable shared practices that do not clearly position any participant as ‘provider’ and ‘recipient’ of support. As Wenger suggested, practicing together formed a community, where newcomers gradually learn about their issues and develop a repertoire of shared resources about how to talk about and share their own and personal issues and vulnerabilities. The community of practice was characterised by being very conventionalised, which reified meaning. Wenger (1998) describes reification as a double‐edged sword, both too little and too much does not give participants enough ‘to play’ with (p. 186). Reification seemed to paradoxically open up new, participatory spaces for collectively negotiating what it could potentially mean to talk with others about personal issues and to re‐negotiate the meaning of personal challenges and how to mitigate or solve them.

A central finding was that many practices at the weekend workshops were concerned with coming together to talk. Learning in the youth program's community of practice on the weekend workshops did not follow a conventional curriculum and although the communicational practices were not specialised, they likely represent a departure from the types of communication the young people were used to at school, with peers and at home. Most of the reified micro‐practices at the weekend workshops created expectations and acceptable opportunities for participants to participate in and learn from talking about and sharing personal emotionally loaded concerns and problems, and to listening and responding to others' concerns and problems.

The participants in the current study did not learn distinctive and specialist skills, such as the craft and entrepreneurialism in the Maker‐community reported in (Bonnette & Crowley, 2020) or the performance skills reported in (Miles, 2007), and this meant that some participants could almost instantly become legitimate central practitioners, while others would take more peripheral positions for longer periods. However, the community did have a hierarchy of expertise. The enrolment into, and later graduation from, the youth programs' young leader training program was regarded as a sign of experienced practice. The youth leaders had significant brokering functions on the boundaries between the particular community of practice and the multiplicities of different social configurations that the young participants belonged to. Wenger (1998) suggested that brokers must often avoid two opposite tendencies: being pulled into become full members and being rejected as intruders. In line with this, in order to be accepted as role models in the youth peer‐led context, the young leaders spoke about their personal problems and their first‐hand experiences of dealing with them (Moensted & Buus, 2022), and the risk of rejection was linked to participants feeling that they were not equal to the young leaders. In this sense, relatability and identification were central elements of negotiated belonging.

Wenger (1998) argued that social learning can form mutually accountable relationships and a sense of belonging to a community where members learn to re‐imagine the world on the basis of their experiences. Unlike the young people observed by Bonnette and Crowley (2020) and Miles (2007), many participants in the current study had formed a strong sense of belonging to the program. We suggest that belonging was stimulated because learning was related to collectively experiencing talking and sharing in a youth‐led community of practice, the reified practices encouraged participants to identify with each other's situations and to imagine a different world for themselves. Concerns and problems were safely negotiated in ‐ and emotionally held by—the community, which added to a strengthened sense of belonging that the young people could draw on as they continued to engage with in other less emotionally intense, parts of the program, for instance weekly goal setting in their local support groups.

Wenger's (1998) theory of communities of practice stated that shared practices can form particular social configurations that are distinct from, and may cut across, other social types of social configurations. In the present study, social learning took place on weekend workshops that strategically brought the young people from disadvantaged backgrounds out of the immediate contexts of their everyday lives and invited them to participate in a different social configuration. Although most of the community practices were non‐specialised, participants only very rarely reported to engage in these practices outside the community. In line with observations by Miles (2007), this strongly indicates that ‘support’ is not linked to the practices learned, they are not easily transferred to other contexts. The program did not change the young person's disadvantaged situation, but formed new identities and a sense of belonging that changed the young person's negotiation of their selves, the meaning of their problems and concerns and the opportunities for change.

### 4.1 Limitations of the study

The selection of Wenger's (1998) theory about learning though collective practices meant that certain data and interpretations of ‘what was going on’ in the youth program were emphasised and others faded into the background and were not reported. We believe that the exploration of the dual process of ‘negotiated meaning’ in the program, participation and reification, was a fruitful perspective that had not been fully explored previously, and at the same time we acknowledge that other perspectives may also have yielded fruitful interpretations, for instance the negotiation of community/group norms. This could have included a valid alternative theoretical perspective with an explicit emphasis on the participants' lived experiences of the program, their explanations of personal and social change or discursive analysis of the speech community, etc.

The majority of interview respondents had been part of the program for several months. We were not able to identify/recruit respondents that had decided or asked to leave the program. This could mean that some potentially more critical perspectives were absent in the findings, for example signs of resisting the program after experiencing community practices as personally unsafe, or experiencing peer‐pressure to add to the shared life events to make them fit group expectations of significant trauma.

Participant observation gave us, the researchers, embodied and personal insight into some of the effects of the program's practices, which would otherwise have been hidden for us. While we were not young people from disadvantaged backgrounds, it provided us with a unique contextual awareness of the participants' statements in formal and informal interviews.

## 5 CONCLUSION

This study contributes to a better understanding of social learning processes in a peer‐led program for young people with disadvantage. Program practices were repetitive and highly conventionalised, which allowed participants to recognise and address their own and others' vulnerabilities and experienced challenges in new, transformational ways. Through the lens of Wenger's (1998) concept ‘negotiated meaning’, peer support was not perceived as something given by a ‘provider’ to a ‘recipient’, but as generated in a community of practice with peers brokering community membership. This invites future research on ‘peer support’ inquiring into situated practices rather than the acquisition of learning and support.

## CONFLICT OF INTEREST

The authors declare that they have no conflict of interest.

## Data Availability

Data available on request from the authors
